# Spontaneous breathing in patients with severe acute respiratory distress syndrome receiving prolonged extracorporeal membrane oxygenation

**DOI:** 10.1186/s12890-019-1016-2

**Published:** 2019-12-09

**Authors:** Jingen Xia, Sichao Gu, Min Li, Donglin Liu, Xu Huang, Li Yi, Lijuan Wu, Guohui Fan, Qingyuan Zhan

**Affiliations:** 1 National Clinical Research Center for Respiratory Diseases, Center for Respiratory Diseases, China-Japan Friendship Hospital, Beijing, China; 2 Department of Pulmonary and Critical Care Medicine, China-Japan Friendship Hospital, Beijing, China; 3 Capital Medical University, Beijing, China; 4 Institute of Clinical Medical Sciences, China-Japan Friendship Hospital, Beijing, China

**Keywords:** Extracorporeal membrane oxygenation, Acute respiratory distress syndrome, Spontaneous breathing, Mechanical ventilation

## Abstract

**Background:**

The use of extracorporeal membrane oxygenation (ECMO) in awake, spontaneously breathing and non-intubated patients (awake ECMO) may be a novel therapeutic strategy for severe acute respiratory distress syndrome (ARDS) patients. The purpose of this study is to assess the feasibility and safety of awake ECMO in severe ARDS patients receiving prolonged ECMO (> 14 days).

**Methods:**

We describe our experience with 12 consecutive severe ARDS patients (age, 39.1 ± 16.4 years) supported with awake ECMO to wait for native lung recovery during prolonged ECMO treatment from July 2013 to January 2018. Outcomes are reported including the hospital mortality, ECMO-related complications and physiological data on weaning from invasive ventilation.

**Results:**

The patients received median 26.0 (15.5, 64.8) days of total ECMO duration in the cohort. The longest ECMO support duration was 121 days. Awake ECMO and extubation was implemented after median 10.2(5.0, 42.9) days of ECMO. Awake ECMO was not associated with increased morbidity. The total invasive ventilation duration, lengths of stay in the ICU and hospital in the cohort were 14.0(12.0, 37.3) days, 33.0(22.3, 56.5) days and 46.5(27.3, 84.8) days, respectively. The hospital mortality rate was 33.3% (4/12) in the cohort. Survivors had more stable respiratory rate and heart rate after extubation when compared to the non-survivors.

**Conclusions:**

With carefully selected patients, awake ECMO is a feasible and safe strategy for severe pulmonary ARDS patients receiving prolonged ECMO support to wait for native lung recovery.

## Background

Extracorporeal membrane oxygenation (ECMO) is often used as a rescue therapy for patients with severe acute respiratory distress syndrome (ARDS) refractory to conventional invasive mechanical ventilation (IMV) [1]. Due to the novel development of ECMO-related technologies [2] and, in 2009, the global outbreak of a new H1N1 influenza virus [3], the number of cases of successful ECMO use for severe ARDS is growing yearly, but the overall mortality rate remains as high as 50–60% [4–7].

“Awake” ECMO is a novel ECMO strategy that uses ECMO for awake, spontaneously breathing and non-intubated critically ill patients [8, 9]. Recently, the application of awake ECMO in patients with chronic end-stage respiratory diseases awaiting lung transplantation [9–12] and chronic obstructive pulmonary disease [13–15] has increased, but its use in severe ARDS patients is rare [16–18]. Several recent clinical physiological studies [19–21] have shown that, in severe ARDS cases, ECMO alone may be able to replace IMV for regulating spontaneous breathing effort and minute ventilation by ECMO gas flow. Therefore, awake ECMO may be a potential novel strategy to replace invasive mechanical ventilation for severe ARDS patients without multiple organ dysfunction [8, 16].

The median duration of ECMO to treat acute respiratory failure is typically 7 to 10 days, however some patients may need longer time to wait for the recovery of native lung function [22, 23]. From Extracorporeal Life Support Organization registry [24], a total of 4488 respiratory failure patients treated with ECMO from 1986 to 2013 were collected, 22% of whom needed ECMO support for more than 14 days (prolonged ECMO), especially since 2008, the number of prolonged ECMO increased markedly. It is still unclear how to wean reasonably from prolonged ECMO in ARDS patients. If ECMO alone can supply adequate gas exchange at this time [19–21], awake ECMO strategy with early extubation in prolonged ECMO patients may reduce the complications associated with invasive positive pressure ventilation, such as barotrauma and VAP, even may improve the prognosis [1]. However, the decision of when to implement awake ECMO is in doubt. Therefore, we first present our experience with 12 ARDS patients treated with awake ECMO to wait for native lung recovery during prolonged ECMO treatment.

## Methods

We retrospectively reviewed the prospectively constituted ECMO database of our 26-bed medical ICU to identify all severe ARDS patients supported with ECMO between July 2013 and January 2018. The screening criteria for awake ECMO patients in the cohort were as follows: (1) had a total ECMO duration of more than 14 days, (2) had endotracheal tube removal at least 48 h before the discontinuation of ECMO. This study was approved by the ethics committee of our center.

Patients were considered for ECMO if they had severe hypoxemia (e.g. the ratio of PaO2 to FiO2 less than 80 mmHg) and uncompensated hypercapnia with academia (pH < 7.15) despite the application of lung protective strategy with high PEEP (12–20 cmH2O) or recruitment maneuver. All the patients in the cohort were received venovenous ECMO (VV-ECMO) with the cannulation of the right or left internal jugular vein and right femoral vein.

Awake ECMO was considered when patients still needed high blood flow ECMO support after 7 to 10 days of ECMO treatment in our center. The final decision to implement awake ECMO was made by multidisciplinary ECMO team. The specific reference standard included the recovery of primary pulmonary disease with decreased pulmonary infiltration on chest x-ray; clear consciousness; strong airway protection capability; no obvious airway secretions or airway inflammation upon fiberoptic bronchoscopy; stable hemodynamics; no arrhythmia; assisted ventilation mode with peak airway pressure < 20 cmH2O, PEEP< 10 cmH2O and FiO2 < 0.5; barotrauma or high risk of barotrauma (such as pulmonary bullae); high risk of VAP, as in the case of immunocompromised patients; ECMO running very well; stable coagulation and fibrinolysis system; no obvious signs of infection of vessel puncture site; and no signs of serious ECMO-related complications, such as major bleeding. The endotracheal intubation was immediately removed once awake ECMO was decided.

The ventilator mode before extubation was pressure support ventilation with positive end-expiratory pressure (PEEP) 7.0 (2.8, 8.0) cmH2O, tidal volume (VT) 3.9 (3.3,6.4) ml/kg, airway peak pressure 18.5 (13.8,23.0) cmH2O, and respiratory rate 21.0(16.2,25.0) bpm. After extubation, the infusion of sedative and analgesic drugs was stopped. Low dose dexmedetomidine was given by 0.5~1 μg/kg/h to maintain the Ramsay score 2~3 points if the patient was agitated. Early mobilization during awake ECMO including active limb activities, sitting on the bed, standing at the bedside was gradually started with the help of intensivists, respiratory therapists, nurses and physical therapists. Besides, respiratory therapists also assisted such patients to practice breathing exercises, such as lung expansion (incentive spirometry), diaphragmatic breathing, directed cough, and airway flutter therapy.

The oxygen therapy modality during awake ECMO could be noninvasive positive pressure ventilation, high-flow nasal cannula, oxygen mask or nasal catheter, according to the patient’s comfort. Awake ECMO treatment was considered failure if patients met the following criteria: severe dyspnea and respiratory rate more than 30 bpm even maximal ECMO support; inability to clear airway secretion; increased pulmonary infiltration on chest x-ray; inadequate gas exchange by ECMO alone; unstable hemodynamics. Once awake ECMO failed, the patients should be reintubated immediately. When native lung function appeared adequate to allow the patient to come off ECMO, we performed a trial off: the blood flow was kept constant, and the ECMO gas source was closed for 2–4 h. When the respiratory rate was < 25 bpm, PaCO2 < 50 mmHg, PaO2 > 60 mmHg and hemodynamic stabilization on spontaneous breathing, the patients were ready for decannulation.

We collected demographic information and data related to ARDS etiology, comorbidity, and pre-ECMO baseline parameters, including Acute Physiology and Chronic Health Evaluation (APACHE) II score, ventilator settings, gas exchange, and the duration of IMV before ECMO. ECMO-related parameters during the ECMO run were also recorded as follows: total ECMO duration, blood flow, gas flow, ventilator settings and ECMO-related complications, including circuit complications (malfunction of any component of the circuit, clot and air in circuit), hemorrhage (gastrointestinal, surgical and pulmonary hemorrhage), ECMO-related infection (VAP, cannula infection and urinary tract infection), barotrauma, neurological complications, renal complications and cardiovascular complications. In addition, we documented the relevant ECMO, respiratory, circulation and routine laboratory test parameters at the onset of and after extubation. The main clinical outcome was hospital mortality, and secondary outcomes included the total duration of IMV and ECMO and the length of stay in the ICU and the hospital.

Data are presented as the mean (SD) or median (range) for continuous variables and as frequency and percentage for categorical variables. Continuous data (respiratory rate and heart rate) were compared between the two groups using Wilcoxon rank sum test. Changes in the measurement of respiratory rate and heart rate between survivor and non-survivor were analyzed using two-way repeated measures ANOVA. A *P* value less than 0.05 level of significance was set. Analyses were performed using SPSS 23 (SPSS, Inc., Chicago, IL, USA).

## Results

### Characteristics of the included patients

A total of 80 patients underwent ECMO due to ARDS during the study period, of which 12 (15%) severe ARDS patients needed prolonged ECMO support (> 14 days) and received awake ECMO (Table 1). Pneumonia was the most common cause of ARDS. The median PaO2/FiO2 ration before ECMO was 61.8 (50.6, 90.4) mmHg. The median time from invasive ventilation to ECMO was 3.2 (1.0,4.7) days. Ventilator settings and gas exchange variables are shown in Table 2.

**Table 1:** Patient characteristics and outcomes

Patient	Cause of ARDS	Underlying disease	Pre-ECMO PaO_2_/FiO_2_ (mmHg)	Pre-ECMO APACHE II Score	Pre-ECMO IMV duration (Days)	Alive
1	Pneumonia (*Legionella*)	Hypertension	50.1	15	1.1	Yes
2	Pneumonia (*Streptococcus pneumoniae*)	None	59.0	11	11.8	No
3	Pneumonia *(H1N1*)	None	50.1	17	3.0	Yes
4	Pneumonia (*H1N1*)	AML	94.7	17	3.5	No
5	Pneumonia (*Pneumocystis carinii*)	SLE	52.0	23	1.0	Yes
6	Pneumonia (*unidentified organism*)	None	96.0	15	0.04	No
7	Pneumonia (*H7N9*)	None	35.2	24	0.4	Yes
8	Acute interstitial pneumonia	None	60.6	17	3.9	Yes
9	Pneumonia (*Aspergillus fumigatus*)	Bronchiectasis	63.0	17	4.8	Yes
10	Acute interstitial pneumonia	None	77.5	13	1.0	Yes
11	Pneumonia (*H1N1*)	None	108.0	10	4.3	Yes
12	Pneumonia (*H1N1*)	Hypertension	65.0	24	7.0	No

*AML* acute myelogenous leukemia; *APACHE II* Acute Physiology and Chronic Health Evaluation II; *IMV* invasive mechanical ventilation; *SLE* systemic lupus erythematosus

**Table 2:** Baseline characteristics and pre-extracorporeal membrane oxygenation ventilator settings and gas exchange variables

Characteristics	Awake ECMO (*n* = 12)
Age (years)	39.1 ± 16.4
Female, n (%)	5 (41.7%)
Body mass index (kg/m^2^)	25.8 ± 5.3
APACHE II score	17.0 (13.5, 21.5)
Murray score	3.4 (2.8, 3.8)
Duration of IMV(day)	3.2 (1.0, 4.7)
Pre-ECMO variables	
VT (ml)	390.0 (216.5, 440.5)
VT pbw (ml/kg)	6.6 (3.3, 7.1)
Respiratory rate (bpm)	30.0 (25.0, 31.0)
Pplat (cmH2O)	31.0 (24.0, 32.5)
PEEP (cmH2O)	14.0 (8.5, 16.5)
FiO2	1.0(1.0, 1.0)
pH	7.40 (7.33, 7.43)
PaCO2 (mmHg)	46.2 (42.9, 52.0)
PaO2/FiO2	61.8 (50.6, 90.4)
Lac (mmol/L)	1.8 (1.4, 2.8)

Definition of abbreviations: *APACHE II* Acute Physiology and Chronic Health Evaluation II, *COPD* chronic obstructive pulmonary disease, *FiO2* fraction of inspired oxygen, *IMV* invasive mechanical ventilation, *PaCO2* Partial pressure of carbon dioxide in arterial blood, *PaO2* Partial pressure of oxygen in arterial blood, *PEEP* positive end-expiratory pressure, *Ppeak* peak airway pressure, *VT* tidal volume, *VT pbw* predicted body weight of tidal volume

### Clinical implementation of awake ECMO

**Fig. 1:**
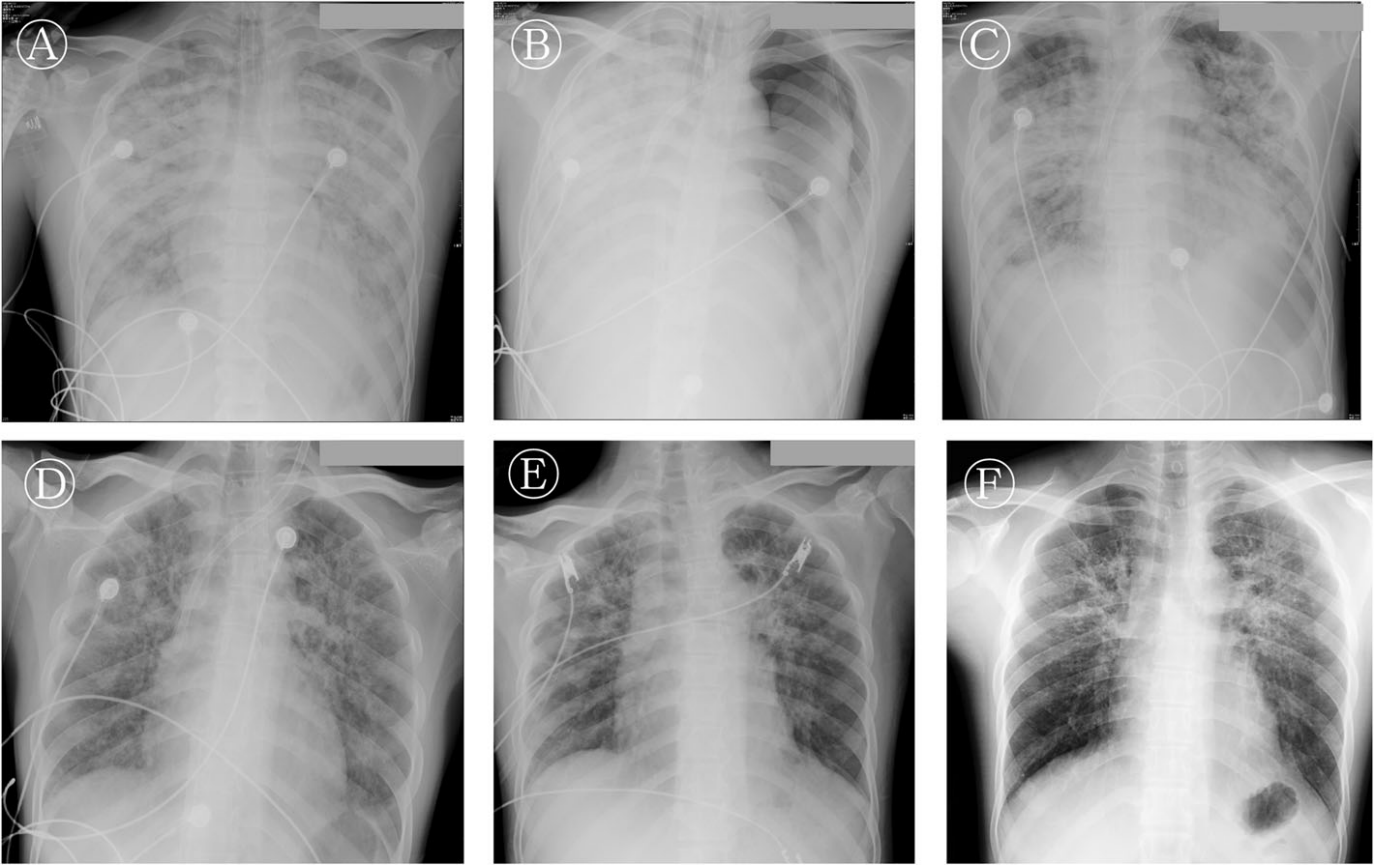
A typical serial chest radiographs of awake ECMO patients (Patient 10). **a**: On ICU admission day. This is the initial chest radiograph after intubation. Increased bilateral opacity in both lungs are noted. **b**: The second day after ICU admission. ECMO insertion was done due to refractory hypoxemia. Chest x-ray shows progressive increase of right opacity and newly left pneumothorax. The left pneumothorax was improved with chest tube and lower mechanical ventilation support level due to ECMO. **c**: On ECMO 5 days. There is improved pulmonary infiltration and pneumothorax, the patient was extubated and underwent awake ECMO to avoid ventilator-associated pneumonia. **d**: On ECMO 30 days. The patient was weaned from ECMO after this chest x-ray. **e**: Fourteen days after weaning from ECMO and discharge. **f**: one month after discharge

In the cohort, patients were extubated after 10.2(5.0, 42.9) days of ECMO with higher ECMO blood flow [3.5(3.2,3.8) L/min] and gas flow [4.3(3.0,7.6) L/min]. Pulmonary infiltration on chest x-ray was decreased significantly (Fig. 1). The choice of oxygen therapy modality for awake ECMO patients was as follows: 8 (66%) patients received oxygen through a high-flow nasal cannula, 2 (17%) patients received conventional oxygen therapy, and 2 (17%) patients were treated with noninvasive positive pressure ventilation. The median duration of awake ECMO was 12.0 (7.0, 26.8) days.

**Fig. 2:**
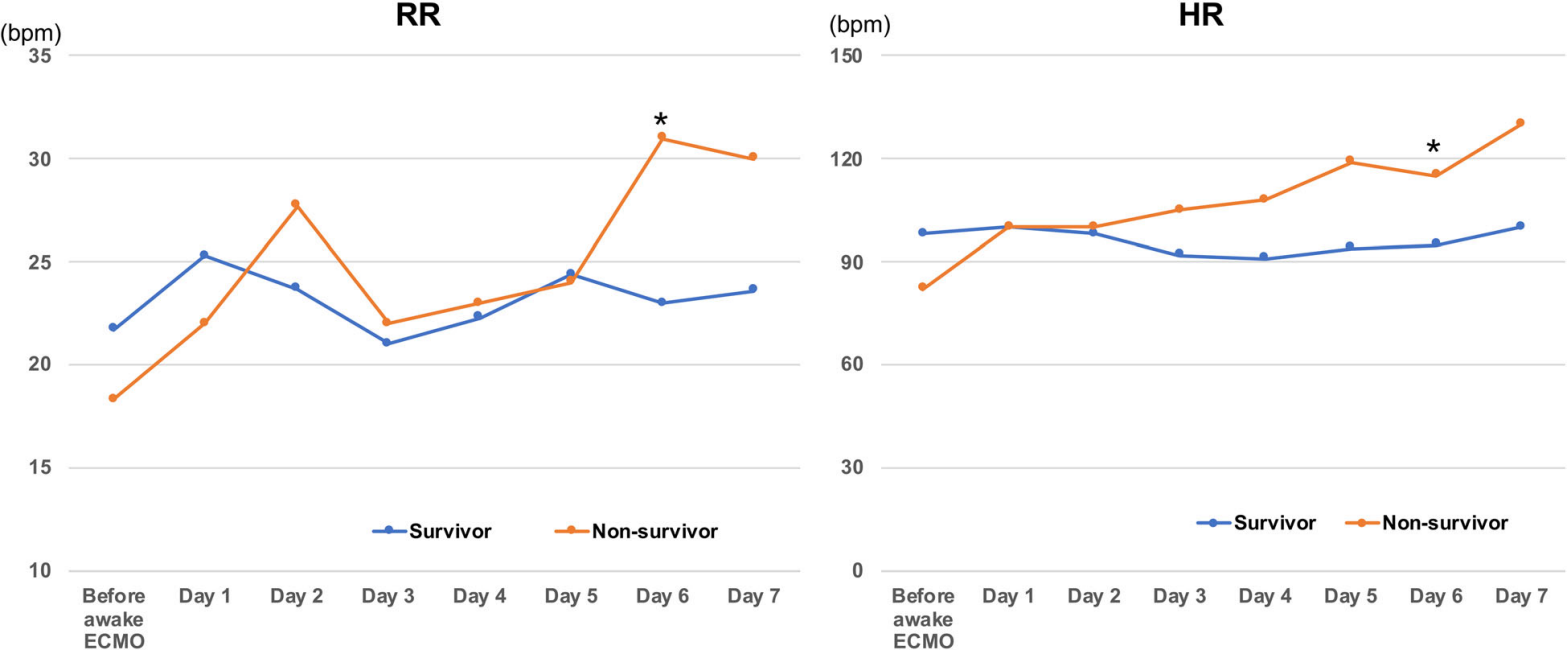
The changes of respiratory rate (left chart) and heart rate (right chart) after extubation in awake ECMO patients. Blue lines represent the survivors with awake ECMO success, yellow lines represent the non-survivors with awake ECMO failure. * *P* < 0.05 between survivor and non-survivor group

After disconnection from invasive ventilation, the respiratory rate increased slightly during the first 24 h both for survivors and non-survivors. In the next 7 days after extubation, respiratory rate (time effect *P* = 0.018, time × group effect *P* = 0.032) and heart rate (time effect *P* = 0.005, time × group effect *P* = 0.001) were more stable in survivors, however, these variables gradually increased in non-survivors. (Fig. 2).

### Outcomes

The hospital mortality was 33.3% (4/12) in the cohort. Three patients (Patient 2, 4 and 6) were reintubated for refractory hypoxemia and severe dyspnea at the seventh, second and fifth days after extubation, and all died of septic shock and multiple organ failure due to the progression of the primary pulmonary disease. Patient 2 was extubated from invasive ventilation after 10 days of ECMO treatment. Due to the progressing of primary pneumonia (*Streptococcus pneumoniae*), patient 2 was reintubated on the seventh day after extubation because of severe hypoxemia (SpO2 85%) and respiratory distress (RR 38 bpm) even with maximal noninvasive positive pressure ventilation (S/T mode, FiO2 1.0) and ECMO support. Patient 4 was removed from tracheal intubation after 29 days of ECMO treatment. As a result of the progressing of *Staphylococcus aureus* pneumonia accompanied by sever empyema and septic shock (BP 90/60 mmHg, Lac 11.7 mmol/L), HFNC (gas flow 50 L/min, FiO2 0.6) was difficult to maintain oxygenation and to relieve dyspnea (RR 35 beats / min) after 2 days of awake ECMO, endotracheal intubation was performed again. Patient 6 started awake ECMO after 7 days of ECMO treatment, and appeared severe hypoxemia (SpO2 88%) and dyspnea (RR 30 bpm) even with NPPV support (continuous positive airway pressure 8 mmHg, FiO2 1.0) on the fifth day of awake ECMO. The patient was finally endotracheally intubated again due to the uncontrolled hospital acquired pneumonia. One patient (Patient 12) successfully underwent awake ECMO at the first time, but severe dyspnea (RR 36 bpm) occurred after removal of the ECMO catheters because of pulmonary embolism, subsequently died of hypovolemic shock and multiple organ failure even ECMO was introduced again.

The total duration of ECMO run in this cohort was 26.0 (15.5, 64.8) days. The maximum ECMO duration was 121 days with a single ECMO membrane when waiting native lung recovery, and the patient survived (Patient 5). The total invasive ventilation duration, lengths of stay in the ICU and hospital were 14.0(12.0, 37.3) days, 33.0(22.3, 56.5) days and 46.5(27.3, 84.8) days, respectively.

### Complications

Pneumothorax (42%, 5 cases) and VAP (33%, 4 cases) were the main ventilator associated-complications before the use of awake ECMO in the cohort. The most common ECMO-associated complications included air in circuit (8%, 1 case), renal replacement therapy (8%, 1case) and ECMO cannula infection (17%, 2 cases), which also had occurred before the start of awake ECMO.

## Discussion

In this retrospective analysis of 12 severe ARDS patients treated with prolonged ECMO, we mainly found that the implementation of awake ECMO in the late stage of ARDS could prompt native lung recovery and would be associated with higher hospital survival rate (66.7%), compared with prior Extracorporeal Life Support Organization registry reported survival rate (45.4%) of prolonged ECMO patients [24]; and awake ECMO did not increase the incidence of ECMO-related complications. It is worth noting that none of the patients with awake ECMO failure survived.

One of the main advantages of awake ECMO lies in the application of ECMO instead of IMV to avoid VAP. ECMO patients are susceptible to VAP due to severe illness, ECMO-induced immunosuppression and a high risk of intestinal microbial translocation [25]. Recently, Grasselli and colleagues [26] found that VAP is the most common type of ECMO-related nosocomial infection; its incidence is as high as 35% or 31.0 cases/1000 ECMO days, and it significantly increased mortality and prolonged the durations of mechanical ventilation, ICU stay and ECMO run. Recently, Rosenberg and colleagues reported that some severe ARDS patients may need more than 2–4 weeks before native lung recovery occurs [22], during which prolonged IMV will inevitably increase the risk of VAP. In our study, the incidence of VAP before awake ECMO was as high as 33%, similar to that reported by Grasselli [26]. Furthermore, 6 (50%) patients needed ECMO support for more than 4 weeks, and the longest duration of ECMO support was 121 days. Therefore, awake ECMO would facilitate early extubation [22] and avoid the risk of VAP for severe ARDS patients requiring prolonged ECMO treatment.

Another important advantage of awake ECMO is the reduced need for sedative and analgesic drugs to facilitate early physical activity and rehabilitative exercise, which can decrease the risk of ICU-acquired weakness [27, 28]. At our center, progressive physical exercises such as sitting on the bed, performing some activities on the bed, and getting out of bed are routinely carried out once awake ECMO is implemented with the help of intensivists, respiratory therapists, nurses and physical therapists, which may also be an important reason for the significant improvement in the prognosis of awake ECMO patients.

Through chest computed tomographic scan, we know that in the late stage of ARDS, the lung tissue is characterized by lower respiratory compliance and high amounts of dead space and is more likely to develop bullae and pneumothorax [29]. Reducing the occurrence of lung injury and promoting lung repair are the focus of ventilation management during the late stage of ARDS [29, 30]. Therefore, for late ARDS patients treated with ECMO, we speculate that when compared with the combination of ECMO and IMV, the early implementation of awake ECMO and extubation may further eliminate lung stress and strain of damaged lung tissue caused by invasive positive pressure ventilation (for example, 42% of awake ECMO patients appeared barotrauma before extubation). However, without invasive ventilatory support, the intensity of spontaneous breathing and transpulmonary pressure will increase, and lung injury in ARDS patients may be aggravated during awake ECMO [1, 8]. Recently, Mauri and colleagues showed that respiratory drive and spontaneous breathing effort during the late stage of ARDS could be adjusted precisely by ECMO gas flow [21]. Therefore, before extubation and awake ECMO, it should be carefully evaluated whether ECMO alone could easily regulate the intensity of spontaneous breathing, and then weigh the risks of high shear stress from increased spontaneous breathing effort [31] after extubation against the risks of ongoing sedative and invasive positive pressure ventilation.

Although awake ECMO has evident above-mentioned clinical benefits, the indications and optimal timing of awake ECMO for severe ARDS remain unclear. Hannover Medical Center first reported the successful use of awake ECMO in the early stage of severe ARDS to avoid intubation in strictly selected patients in case reports [16, 17]. In addition, Yeo and colleagues [18] used awake ECMO as a weaning strategy in 10 patients with severe postoperative ARDS and found that 70% of the patients were successfully weaned from IMV and ECMO, the mean ECMO duration was only 9.1 ± 2.2 days. Be different from these researches, we tried to explore the clinical advantages of awake ECMO at the late stage of severe ARDS receiving prolonged ECMO treatment [median ECMO duration 26.0 (15.5, 64.8)]. In the early stage of the disease, to attenuate lung injury and reduce venous admixture, positive pressure ventilation is often required to open the alveoli and keep them open. Additionally, most severe ARDS patients need artificial airways for sputum drainage, airway protection and infection control. In fact, control ventilation mode in the early stage of severe ARDS may be more important than assisted ventilation mode to improve clinical outcomes [32]. Therefore, we think that awake ECMO could be considered as soon as possible if severe ARDS patients treated with prolonged ECMO meet the following indications, especially in the case of patients with a high risk of barotrauma and VAP: the recovery of pneumonia; consciousness and good airway protection capacity; a moderate respiratory support level with airway peak pressure < 20 cmH2O, PEEP< 10 cmH2O and FiO2 < 0.5; ECMO blood flow < 4 L/min; satisfactory gas exchange; and no serious ECMO-related complications. Regarding the type of oxygen therapy during the awake ECMO strategy, high-flow nasal cannula may be the best choice. A recent study confirmed that high-flow nasal cannula can effectively prevent respiratory failure and reintubation after extubation in acute respiratory failure patients [33]. In this study, 67% of the awake ECMO patients were supported by a combination of ECMO and high-flow nasal cannula.

In addition to careful case selection, maintaining the ECMO run carefully and avoiding ECMO-related severe complications are also important prerequisites for the successful implementation of awake ECMO. There was no obvious increase in ECMO-related complications in our patients, which may be explained by the promising advancement of ECMO-related equipment and technology, such as low-resistance gas exchange membranes, centrifugal blood pumps, heparin-coated catheters and improved intravascular catheters, as well as advancements in the management of critical illness [2, 34].

This study has several limitations. First, the number of patients was small, and all patients were selected from a single center. Second, this study was not prospectively designed, and the possibility of patient selection and intervention biases cannot be excluded. Finally, awake ECMO was implemented after median 10.2 days of ECMO, and the results of the study cannot indicate whether earlier implementation of awake ECMO would further improve the clinical outcomes; therefore, a prospective clinical study is required to explore the best timing of awake ECMO implementation during prolonged ECMO treatment.

## Conclusions

Our data suggest that awake ECMO is a feasible and safe strategy for carefully selected pulmonary ARDS patients receiving prolonged ECMO support to wait for native lung recovery. Comparative analyses of awake ECMO and non-awake ECMO are required to prospectively investigate the optimal ECMO weaning strategy for prolonged ECMO patients.

## Data Availability

The data that support the findings of this study are available from the corresponding author upon reasonable request.

## Abbreviations

APACHE II: Acute Physiology and Chronic Health Evaluation II
ARDS: Acute respiratory distress syndrome
ECMO: Extracorporeal membrane oxygenation
IMV: Invasive mechanical ventilation
PEEP: Positive end expiratory pressure
VAP: Ventilator-associated pneumonia
VILI: Ventilator-induced lung injury
VV-ECMO: Venovenous extracorporeal membrane oxygenation
